# Specific MRI Abnormalities Reveal Severe Perrault Syndrome due to CLPP Defects

**DOI:** 10.3389/fneur.2016.00203

**Published:** 2016-11-16

**Authors:** Tom E. J. Theunissen, Radek Szklarczyk, Mike Gerards, Debby M. E. I. Hellebrekers, Elvira N. M. Mulder-Den Hartog, Jo Vanoevelen, Rick Kamps, Bart de Koning, S. Lane Rutledge, Thomas Schmitt-Mechelke, Carola G. M. van Berkel, Marjo S. van der Knaap, Irenaeus F. M. de Coo, Hubert J. M. Smeets

**Affiliations:** ^1^Department of Clinical Genetics, Maastricht University Medical Centre, Maastricht, Netherlands; ^2^Department of Genetics and Cell Biology, School for Oncology and Developmental Biology, Maastricht University Medical Centre, Maastricht, Netherlands; ^3^Maastricht Centre for Systems Biology (MaCSBio), Maastricht, Netherlands; ^4^Department of Neurology, Erasmus MC, Rotterdam, Netherlands; ^5^Department of Neurology and Genetics, University of Alabama at Birmingham, Birmingham, AL, USA; ^6^Department of Neuropediatrics, Luzerner Kantonsspital, Kinderspital, Luzern, Switzerland; ^7^Department of Child Neurology, Neuroscience Campus Amsterdam, VU University Medical Center, Amsterdam, Netherlands

**Keywords:** genetic diagnosis, brain-MRI, Perrault syndrome type 3, Perrault syndrome, *CLPP*

## Abstract

In establishing a genetic diagnosis in heterogeneous neurological disease, clinical characterization and whole exome sequencing (WES) go hand-in-hand. Clinical data are essential, not only to guide WES variant selection and define the clinical severity of a genetic defect but also to identify other patients with defects in the same gene. In an infant patient with sensorineural hearing loss, psychomotor retardation, and epilepsy, WES resulted in identification of a novel homozygous *CLPP* frameshift mutation (c.21delA). Based on the gene defect and clinical symptoms, the diagnosis Perrault syndrome type 3 (PRLTS3) was established. The patient’s brain-MRI revealed specific abnormalities of the subcortical and deep cerebral white matter and the middle blade of the corpus callosum, which was used to identify similar patients in the Amsterdam brain-MRI database, containing over 3000 unclassified leukoencephalopathy cases. In three unrelated patients with similar MRI abnormalities the *CLPP* gene was sequenced, and in two of them novel missense mutations were identified together with a large deletion that covered part of the *CLPP* gene on the other allele. The severe neurological and MRI abnormalities in these young patients were due to the drastic impact of the *CLPP* mutations, correlating with the variation in clinical manifestations among previously reported patients. Our data show that similarity in brain-MRI patterns can be used to identify novel PRLTS3 patients, especially during early disease stages, when only part of the disease manifestations are present. This seems especially applicable to the severely affected cases in which CLPP function is drastically affected and MRI abnormalities are pronounced.

## Introduction

For identification of the genetic causes of clinically and genetically heterogeneous neurological disease, whole exome sequencing (WES) has become the prime method of investigation as a rapid, single test. Especially for conditions that can be caused by a large number of candidate genes, WES provides an unbiased approach to identify the genetic cause, precluding comprehensive analysis of all individual genes (1–4). By contrast, in some neurological conditions, clinical symptoms or additional investigations may directly point to one or a few candidate genes, enabling faster and cheaper diagnosis by targeted gene analysis (5–7). In practice, clinical characterization and WES go hand-in-hand (8, 9). Clinical data are essential not only to guide WES variant selection but also to define the impact and severity a genetic defect may have.

We investigated a patient in whom MRI and WES studies enforced each other in interpreting the genetic defect and establishing a diagnosis. We found that in this case MRI data are predictive for the severity of the clinical manifestations and can be used to identify severe cases of Perrault syndrome due to *CLPP* mutations.

## Materials and Methods

### Study Design

We performed WES on a patient (patient 1.1) from consanguineous parents who suffered from pronounced neurological problems and had specific abnormalities on brain-MRI, comparable to his affected sibling (patient 1.2). Subsequently, the Amsterdam brain-MRI database containing over 3000 unclassified leukoencephalopathy cases was checked manually by experts to identify patients with a similar MRI pattern. This resulted in selection of three unrelated patients (patient 2, patient 3.1, and patient 4) who were then, together with their affected siblings (patient 3.2), subject to targeted *CLPP* gene analysis.

### METc

Procedures involving human participants were in accordance with the ethical standards of the institutional (MUMC+, VUmc) research committee. All subjects gave written informed consent in accordance with the Declaration of Helsinki.

### Patients

The clinical details of the patients with *CLPP* mutations were retrospectively reviewed.

### Magnetic Resonance Imaging

For all patients, at least one MRI was available for review. The studies had been performed on different MRI machines of different vendors, precluding quantitative measurements, but allowing MRI pattern analysis. In all patients, axial T2-weighted images were used for systematic analysis of the abnormalities (10). Based on the MRI findings in the first patient, the MRI criteria for further inclusion of patients were signal abnormalities in the deep and subcortical cerebral white matter, signal abnormalities in the middle blade of the corpus callosum, and absence of abnormalities in basal nuclei and thalami.

### Homozygosity Mapping

Peripheral blood DNA was prepared and labeled for Affymetrix Human Mapping 250 K array according to the manufacturer’s protocol (Affymetrix GeneChip, Santa Clara, CA, USA).

### WES Analysis

Agilent SureSelect version 4 (Agilent Technologies, Santa Clara, CA, USA) was used for exome enrichment, and massive parallel sequencing was performed on an Illumina HiSeq2000 platform, using a 2 × 100 bp paired end setting (Illumina, San Diego, CA, USA). Exome data of proband 1.1 were filtered for homozygous variants in homozygosity regions. Only variants with allele frequencies lower than 1% (dbSNP137), consisting of non-synonymous substitutions, INDELs (in-frame and frameshift), nonsense mutations, and splice-variants were evaluated. Non-annotated variants were maintained, unless allele frequencies exceeded 5% prevalence in our in-house patient database. Enrichment for missense variants in conserved protein domains was ensured by removal of variants with PhyloP values <3.5. Pathogenicity of non-synonymous missense mutations was estimated by PolyPhen-2 (11), SIFT (12), PROVEAN (13), and Mutation Taster (14).

### Sanger Sequencing and Paternity Testing

*CLPP* exons and flanking intronic regions were PCR amplified using M13-tailed primers (Table S1 in Supplementary Material) and sequenced by the ABI 3730 automatic sequencer (Applied Biosystems, Foster City, CA, USA). *CLPP* Sanger sequencing was performed in all patients, sibs, and parents. Paternity was tested by multiplex VNTR analysis using the AmpFISTR Identifiler PCR amplification kit according to manufacturer’s manual (Applied Biosystems, Foster City, CA, USA).

### *CLPP* mRNA Expression

RNA was isolated from patient and control skin fibroblasts using the MagMAX-96 Total RNA Isolation Kit (ThermoFisher scientific, Waltham, MA, USA). cDNA was synthesized by qScript cDNA Synthesis Kit (Quanta Biosciences, Gaithersburg, MD, USA) using 1 μg RNA input. mRNA expression was measured by the 7900HT Fast Real-Time PCR System (Applied Biosystems, Foster City, CA, USA) based on a primer pair at the 5′ side (exon 2 and 3, 104-bp amplicon) and 3′ side (exon 5 and 6, 170-bp amplicon) of the *CLPP* transcript (NM_006012). *CLPP* expression was normalized against the *TBP* housekeeping gene (TATA box-binding protein, NM_003194, primer set in exon 5 and 6, 89-bp amplicon) (Table S1 in Supplementary Material). Sensimix Sybr Hi-Rox (Bioline, Taunton, MA, USA) was used for cDNA amplification, and a relative *CLPP* mRNA expression was calculated using the 2^−ΔΔCt^ method, normalizing to healthy control fibroblasts. Statistical significance was calculated using a *T*-TEST (one-tailed, two-sample equal variance) with Bonferroni correction.

### Protein Structure Modeling

An X-ray structure of the human mitochondrial CLPP heptamer (PMID: 15522782) at 2.10 A resolution has been used for visualizing mutations in the protein structure. The structure was displayed in PyMol (ver.1.8), and mutations were introduced with the Mutalizer package.

### mtDNA and *CLPP* Copy Number Quantification

mtDNA copy number was quantified by comparing mtDNA encoded *ND5* copy number (mtDNA NADH dehydrogenase 5, NC_012920.1) with the nuclear *B2M* gene (nuclear gene beta-2-microglobulin, NC_000015.10) (Table S1 in Supplementary Material). DNA amplification was performed under similar conditions as reported above. A relative mtDNA copy number was defined using the 2^−ΔΔCt^ method, normalizing to heathy control fibroblasts. Statistical significance was calculated using a *T*-TEST (one-tailed, two-sample equal variance) with Bonferroni correction.

A similar method was used to determine the *CLPP* nDNA copy number (NC_000019.10). A primer pair located in intron 2 and exon 3 (primer set exon 3, 176-bp amplicon) and in intron 5 and exon 6 (primer set exon 6, 168-bp amplicon) of the *CLPP* gene were used. Two amplicons were designed to cover part of exon 4, a primer pair in intron 3 and exon 4 (primer set 4A; 135bp amplicon), and exon 4 and intron 4 (primer set 4B; 164bp amplicon) (Table S1 in Supplementary Material).

## Results

### Patients

Table S2 in Supplementary Material contains the clinical details of all patients. The proband, patient 1.1 (born in 1993), was the second child from consanguineous parents. He presented with congenital sensorineural deafness, psychomotor retardation, epilepsy, and small stature. He had a younger brother, patient 1.2 (born in 1995), with a similar clinical picture. Patient 2 (born in 1994) and sibling-patients 3.1 and 3.2 (born in 1989 and 1994, respectively) also had early onset deafness, stunted growth, developmental retardation of variable severity, and motor problems due to ataxia, spasticity, and peripheral neuropathy. Two of the patients had epilepsy. In all patients, the disease course was slowly progressive and in some of them provoking factors like febrile infections could lead to episodes of regression.

### Magnetic Resonance Imaging

**Figure 1:**
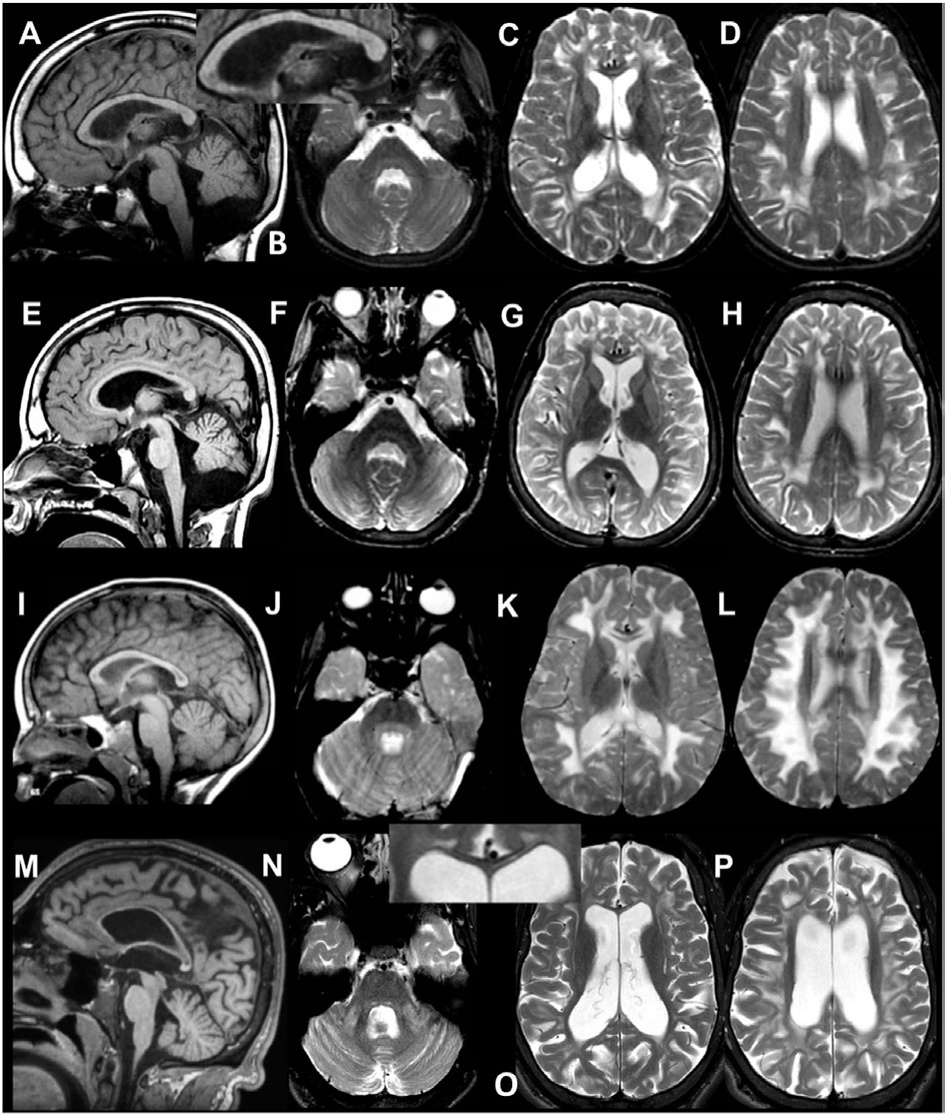
**MRI of patient 1.1 at 9 years (A–D) and 12 years (E–H)**. The first MRI shows signal abnormalities in the middle blade of the corpus callosum [**(A,C)** and inset in **(A)**]; inhomogeneous mainly multifocal signal abnormalities in the subcortical and deep cerebral white matter, sparing a periventricular rim **(C,D)**; and slight cerebral **(C,D)** but no cerebellar atrophy **(A,B)**. At follow-up, slightly increased cerebral and cerebellar atrophy is seen. **MRI of patient 2 at 4 years (I–L) and 20 years (M–P)**. The first MRI shows signal abnormalities in the middle blade of the corpus callosum **(I,K)**, inhomogeneous, mainly confluent signal abnormalities in the subcortical and deep cerebral white matter, sparing a periventricular rim **(K,L)** and no cerebral or cerebellar atrophy **(I–L)**. At follow-up, severe cerebral and milder cerebellar atrophy is seen **(M–P)**. The corpus callosum is highly atrophic, but the selective involvement of the middle blade is still seen [inset in **(O)**]. Within the posterior fossa, signal abnormalities are present in the middle cerebellar peduncles and pontine central tegmental tracts **(N)**.

Table 1 contains the MRI details of all patients, also illustrated in Figure 1. All patients had inhomogeneous, partially multifocal, and partially confluent signal abnormalities in the deep and subcortical white matter, largely sparing the directly periventricular white matter. In all patients, the middle blade of the corpus callosum was affected, while the inner and outer blades were spared. Only one patient (patient 2) showed signal abnormalities in the cerebellar white matter and central tegmental tracts in the pons, while two patients had signal abnormalities in the middle cerebellar peduncles. Over time, cerebral and less severe cerebellar atrophy occurred.

**Table 1:** **MRI findings**.

Patient number	Patient 1.1	Patient 1.2	Patient 2	Patient 3.1	Patient 3.2

Year of birth	1995	1993	1994	1989	1994

MRIs (ages in years)	2 (9, 10, 12)	1 (8)	4 (3, 11, 12, 17, 20)	2 (11, 20)	1 (4)
**First MRI**					
Cerebral WM abn.^a^, aspect	Inhomogeneous, partially multifocal	Multifocal	Inhomogeneous, partially multifocal	Inhomogeneous, partially multifocal	Inhomogeneous, partially multifocal
Predominance cerebral WM abn.	Deep + subcortical	Deep	Deep + subcortical	Deep + subcortical	Deep + subcortical
Sparing cerebral WM	Periventricular	Periventricular + subcortical	Periventricular	Periventricular	Periventricular
Corpus callosum genu abn.	+, esp. middle blade	+, middle blade	+, esp. middle blade	+, esp. middle blade	+, esp. middle blade
Corpus callosum body abn.	−	−	−	−	−
Corpus callosum splenium abn.	+	+, middle blade	+	−	+
Internal/external capsule abn.	−/−	−/−	−/−	−/−	−/−
Swelling of abn. WM	−	−	−	−	−
Rarefaction of abn. WM^b^	−	−	+	−	−
WM cysts^c^	−	−	−	−	−
Cerebral cortex signal abn.	−	−	−	−	−
Basal nuclei signal abn.	−	−	−	−	−
Thalamus signal abn.	−	−	−	−	−
Cerebral atrophy	Slight	−	−	Slight	−
Cerebellar WM signal abn.	−	−	+	−	−
Hilus dentate nucleus abn.	−	−	−	−	−
Cerebellar cortex signal abn.	−	−	−	−	−
Dentate nucleus signal abn.	−	−	−	−	−
Cerebellar atrophy	−	−	−	−	−
Middle cerebellar peduncles abn.	−	+	+	−	−
Brainstem signal abn.	−	−	+, central tegmental tracts	−	−
Brain stem atrophy	−	−	−	−	−
Contrast enhancement	−	n.d.	n.d.	n.d.	n.d.
Restricted diffusion^d^	n.d.	n.d.	n.d.	n.d.	n.d.
Elevated lactate in MRS	−	n.d.	n.d.	n.d.	n.d.
**Latest MRI**					
Change over time	Slight further cerebral atrophy		Cerebral and cerebellar atrophy; no diffusion restriction; no contrast enhancement	Cerebral and cerebellar atrophy, cerebellar WM abn., no diffusion restriction	−

*^a^Signal abnormalities defined as an abnormally high signal on T2-weighted images*.

*^b^White matter rarefaction defined as T2-hyperintense WM with low signal on FLAIR, but not as low as cerebrospinal fluid*.

*^c^Cysts defined as T2-hyperintense WM with low signal on FLAIR, as low as cerebrospinal fluid*.

*^d^Restricted diffusion assessed using the apparent diffusion coefficient (ADC) to avoid T2-shine-through effects*.

*WM, white matter; abn., abnormalities/abnormal; MRS, magnetic resonance spectroscopy; n.d., not done; esp., especially; +, present; −, absent; FLAIR, fluid-attenuated inversion recovery*.

Patient 4, who did not have *CLPP* mutations, had more extensive cerebral white matter abnormalities at disease onset and striking swelling of the corpus callosum. In the course of a few years, the white matter signal abnormalities largely disappeared, although the middle blade of the corpus callosum remained abnormal. He developed very mild atrophy.

### WES and *CLPP* Analysis

**Figure 2:**
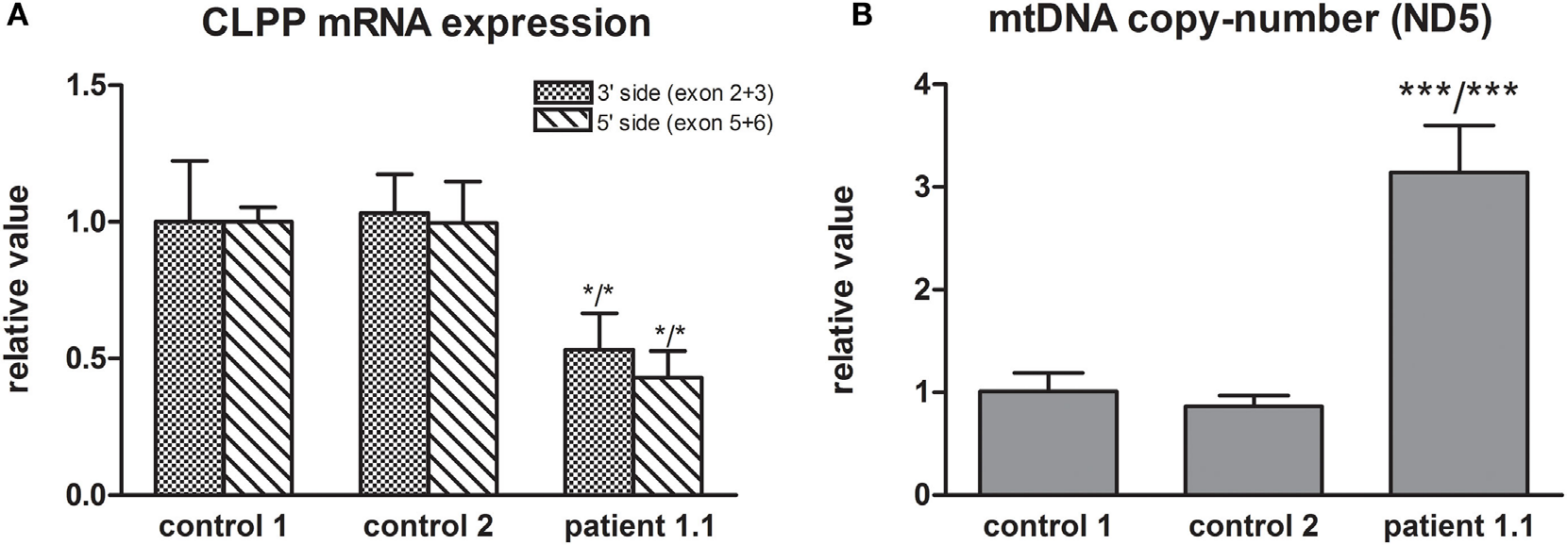
**(A)** CLPP mRNA expression was measured using a primer pair at the 3′ side (exon 2 and 3) and 5′ side (exon 5 and 6) of the transcript (NM_006012) and was normalized to TBP. Approximately 50% lower CLPP mRNA levels were found in patient 1.1 fibroblasts compared to controls. **(B)** mtDNA copy number was measured based on ND5 copy number and was normalized to nuclear B2M. Approximately a threefold copy number increase was measured in patient 1.1 (proband) fibroblasts compared to control fibroblasts.

Whole exome sequencing of patient 1.1 (family 1) resulted, after filtering, in 34 homozygous variants. His brother, patient 1.2, was affected as well and homozygosity mapping showed a 56-Mb overlap between the siblings, resulting in 19 candidate genes. Candidate mutations were further limited to evolutionary conserved protein positions. The most promising mutation was a novel (non-annotated) homozygous mutation in a highly conserved domain of the *CLPP* gene (NM_006012_c.21delA, chr: 19, g.6361606delA). The single nucleotide deletion is located in exon 1 and is expected to cause a frameshift with a nonsense mutation in exon 3 [p.(Ala10Profs*117)]. The resulting “stop” mutation would cause a loss of 160 amino acids of the evolutionary highly conserved CLPP protein and is likely to result in nonsense mediated mRNA decay. Indeed, *CLPP* mRNA expression was decreased to approximately 50% in patient fibroblasts compared to control fibroblasts (Figure 2A), confirming a role for nonsense-mediated decay. As it has been reported in mice studies that *CLPP* mutations affect mtDNA copy number, we determined this in fibroblast DNA from patient 1.1 (15). Patient 1.1 showed an approximately threefold increase in mtDNA copy number (Figure 2B). Mutation segregation testing in family 1 indicated that the unaffected parents and sister were heterozygous, and the affected brother was homozygous for the variant.

**Figure 3:**
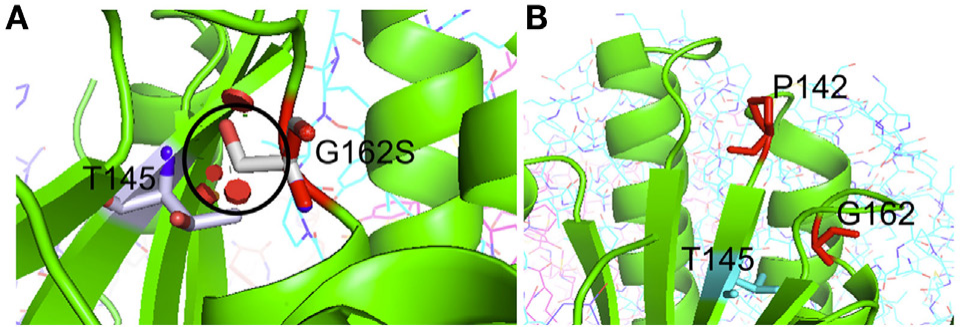
**Crystal structure-based *in silico* modeling of the CLPP protein (Uniprot accession number: Q16740)**. **(A)** p.(Gly162Ser) introduces a long serine side chain that interacts with the Thr145 amino acid and destabilizes the interaction between the alpha-helix structure (residues 154–160) and beta sheet (residues 143–152). Red disks indicate significant van der Waals overlap, including a conflict with a p.145 position previously reported in CLPP patients. **(B)** p.(Pro142Leu) changes a proline residue, located in a loop (139–142) at the boundary with a beta sheet (143–152), into a highly hydrophobic Leucine residue. This drastic hydrophobicity change could interfere with CLPP–CLPX binding at the interface.

**Figure 4:**
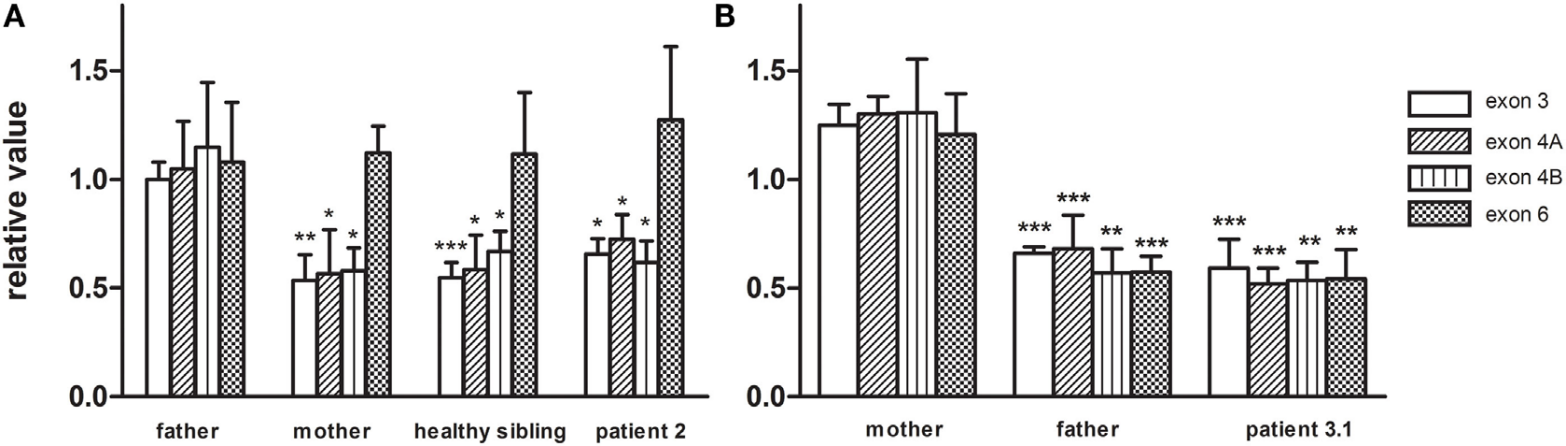
**The CLPP nDNA copy number was determined based on amplicons in exon3, exon 4, and exon 6 and was normalized to B2M**. Copy numbers were relative to control DNA. **(A)** In family 2, CLPP copy numbers of patient 2, the healthy sibling, and mother were compared to the heterozygous father and indicated a maternally inherited CLPP deletion. **(B)** In family 3, CLPP copy numbers of patient 3.1 and the father were compared to the heterozygous mother and indicated a paternally inherited CLPP deletion.

Patient 2 (family 2) carried an apparently homozygous c.484G > A (NM_006012.2_ c.484G > A; chr: 19; g.6364579G > A) missense mutation in exon 4, causing an amino acid substitution at p.(Gly162Ser), which is located at the border of a helix domain (p.154–160; Nextprot: EC 3.4.21.92). The mutation affects an evolutionary conserved protein domain (PhyloP: 5.61) and was predicted harmful at the protein level (SIFT: 0.000, PROVEAN: −5.8, MutationTaster: disease causing). To examine the impact of the p.(Gly162Ser) amino acid substitution on CLPP protein structure, template-based *in silico* protein modeling was performed based on the crystal structure of the CLPP heptamer (UNIPROT accession number: Q16740, Protein Data Bank identifier 1tg6). The p.(Gly162Ser) mutation in our patient introduces a bulky serine side chain that destabilizes the interaction between two structural domains, the alpha-helix (residues 154–160) and the beta sheet (residues 143–152) (Figure 3A). More importantly, the serine side chain protrudes toward and clashes with the Thr145 amino acid of the beta sheet, a residue in which the p.(Thr145Pro) mutation, resulting in significant disruption of the protein structure in the region, has been reported as disease causative (16). Segregation analysis in the family indicated that patient 2 was hemizygous for the mutation, as the father was heterozygous carrier and the mother did not carry the mutation (wild-type). To test for the presence of a maternal deletion, we determined the *CLPP* copy number for regions in exon 3, 4, and 6 in the patient and his parents. The patient’s copy number was approximately 50% lower for exon 3 and exon 4 compared to the father (Figure 4A). The same decrease in copy number was seen in DNA from the mother and healthy sibling, indicating that the deletion was inherited from the mother. Interestingly, the copy number of exon 6 did not significantly differ, which is in line with the presence of a heterozygous SNP in exon 6 of the patient. The maternally inherited deletion is therefore expected not to exceed exon 6.

In patient 3.1 and his affected brother patient 3.2 (family 3), an apparently homozygous c.425C > T (NM_006012.2_ c.425C > T; chr: 19; g.6364520C > T) missense mutation was identified in exon 4, in an evolutionary highly conserved protein domain (PhyloP: 5.69). It was predicted to have highly pathogenic impact on protein function (SIFT: 0.003, PROVEAN: −8.9, MutationTaster: disease causing). The resulting p.(Pro142Leu) amino acid substitution is located at the border of a Beta-strand (p.143–152; Nextprot: EC 3.4.21.92), near two mutations [p.(Thr145Pro) and p.(Cys147Ser)] that have been included in the HGMD database for disease-causing mutations. The p.(Pro142Leu) mutation changes a non-reactive proline residue, located in a loop (139–142) at the boundary with the beta sheet (143–152), into a highly hydrophobic leucine residue (Figure 3B). This loop is localized at the CLPP–CLPX interface that is mediated by the beta sheet structure shown to be affected by the previously reported Thr145Pro mutation and p.(Gly162Ser) mutation, reported here for patient 2. Likely, the drastic hydrophobicity change, caused by the p.(Pro142Leu) mutation, will affect the efficiency of CLPP–CLPX docking due to changes at the CLPP–CLPX interface, a region in which mutations have previously been related to pathogenicity (16). Segregation testing showed that his affected sibling carried the same mutation and that both patients were hemizygous, since only the mother was a heterozygous carrier, whereas the father did not carry the mutation. Non-paternity was excluded. *CLPP* copy numbers for regions in exon 3, 4, and 6 were determined for patient 3.1 and his parents (Figure 4B). An approximately 50% decrease in copy number of exon 3, exon 4, and exon 6 was observed in the patient and his father, but not his mother. The genomic deletion, which is transmitted by the father, is therefore expected to cover at least exon 3 to exon 6 of the *CLPP* gene.

In patient 4, no mutations were identified in the *CLPP* gene.

## Discussion

Using WES, we identified a novel homozygous single nucleotide deletion (c.21delA) in the *CLPP* gene of two affected siblings of family 1. The resulting frameshift was expected to introduce a premature stop codon in exon 3. Quantification of *CLPP* transcript expression in patient fibroblasts showed an approximately 50% decrease in *CLPP* mRNA levels, indicating nonsense-mediated decay. CLPP plays a role in the mitochondrial unfolded protein response pathway (mtUPR) by multimerizing with the CLPX chaperone within the mitochondrial matrix, thereby forming a proteasome-like cylinder that is capable of hydrolyzing mitochondrial proteins into small peptides. It is therefore expected to react upon protein conformational stress in mitochondria by inducing mitochondrial stress signaling pathways (17, 18). Molecular characterization of *Clpp* null mice has demonstrated that the absence of *Clpp* transcripts leads to significant increases in mtDNA copy number in several tissues (testis, ovary, heart, and brain) (15). We confirmed an approximately threefold increase in mtDNA copy number in fibroblasts of patient 1.1, indicating that the c.21delA mutation was likely to affect CLPP function in the same way.

We used the brain-MRI of patient 1.1 as a template to screen an Amsterdam brain-MRI database containing over 3000 unclassified leukoencephalopathy for comparable characteristics and selected three patients based on similarities in brain-MRI and clinical manifestations for targeted *CLPP* sequencing (19). In two of these patients, we identified harmful missense mutations in *CLPP*. In the third patient, no CLPP mutation was identified. This patient initially fulfilled the MRI criteria, but over the years, the white matter abnormalities improved and largely disappeared without significant cerebral or cerebellar atrophy. A p.(Gly162Ser) mutation in patient 2 and p.(Pro142Leu) mutation in patient 3.1 and 3.2 were located at an evolutionary highly conserved amino acid position and were predicted to be pathogenic according to several *in silico* prediction tools. Crystal structure-based protein modeling showed that the p.(Gly162Ser) mutation is likely to destabilize the interaction between an alpha-helix structure and beta sheet and could therefore disrupt CLPP protein structure. The mutation interferes with the amino acid p.Thr145, a residue in which a disease-causing mutation was previously reported in a patient with pronounced neurological symptoms (Table 2). Also, changes in hydrophobicity of the p.(Pro142Leu) substitution, located adjacent to this beta sheet, could interfere with the CLPP–CLPX binding-interface (20). Changes at the CLPP–CLPX interface have previously been suggested to be involved in disease (16). *CLPP* copy number quantification in fibroblasts of patient 2 and patient 3.1 showed that both mutations were actually hemizygous. Whereas the *CLPP* deletion in patient 2 was maternally inherited and did not exceed exon 6, the deletion in patient 3.1 was paternally inherited and covered at least exon 1 to 6. Our data indicate that large deletions can be responsible for a substantial part of the inherited CLPP defects and that one should be aware that these mutations could be missed in purely sequencing-based approaches.

**Table 2:** **Indicates the neurological implications of CLPP mutations in our patients (family 1–3) and earlier reported CLPP cases (family 4–10)**.

Family	Pronounced neurological symptoms	White matter abnormalities (MRI)	Mutations
1 consanguineous (patient 1.1 and 1.2)	Yes	Yes	NM_006012_c.21delA (homozygous) frameshift with nonsense mutation NMD: 60% transcript loss
2 non-consanguineous (patient 2)	Yes	Yes	NM_006012.2_c.484G > A p.(Gly162Ser) (hemizygous) large allelic CLPP deletion
3 non-consanguineous (patient 3 and 3.1)	Yes	Yes	NM_006012.2_c.425C > T p.(Pro142Leu) (hemizygous) large allelic CLPP deletion
**Literature reported**			
4 consanguineous (3 patients)	Yes; the 8-month-old boy did not show signs of PRLTS3 yet	Yes (eldest sibs)	c.685T > G p.(Tyr229Asp) (homozygous)
5 consanguineous (3 patients)	Yes	Yes (single patient analyzed)	c.433A > C p.(Thr145Pro) (homozygous)
6 consanguineous (4 patients)	No	n.d.	c.440G > C p.(Cys147Ser) (homozygous)
7 consanguineous (2 patients)	No	n.d.	c.439T > A p.(Cys147Ser) (homozygous)
8 consanguineous (3 patients)	No	n.d.	c.270p(+)4A > G (homozygous) splice donor site mutation weakens donor splice site function
9 consanguineous (1 patient)	No	No	c.430T > C p.(Cys144Arg) (homozygous)
10 unknown (ahead of print) (2 patients)	No	n.d.	c.624C > G p.(Ile208Met) (homozygous)

Our patients, who were all males, suffered from heterogeneous manifestations, involving congenital sensorineural hearing loss (SNHL), psychomotor retardation, ataxia, autism, epilepsy, and short stature. Comparable symptoms have previously been reported in seven other families with *CLPP* defects (Table 2) (16, 21–24). Apart from the consistent involvement of SNHL, sex-related clinical features have been reported such as female ovarian dysgenesis and infertility due to azoospermia in males (15, 22). Our male patients were not tested for infertility. Consequently, *CLPP* mutations cause highly heterogeneous symptoms that have been classified as Perrault syndrome type 3 (PRLTS3). T2-weighted brain-MRI of our patients showed inhomogeneous signal abnormalities in the deep and subcortical cerebral white matter and middle blade of the corpus callosum, with over time severe cerebral atrophy. However, neurological implications are not always pronounced in PRLTS3. Whereas two earlier reported families showed severe neurological symptoms and white matter abnormalities on MRI, other families showed a much milder phenotype. Previously, Jenkinson et al. explained differences in clinical impact by the more drastic structural protein changes caused by the p.(Thr145Pro) mutation in comparison to p.(Cys147Ser). The c.270 + 4A > G splice site mutation was shown to cause only partial ablation of donor splice site function and was therefore likely to have residual CLPP activity (Table 2) (16). Our data support the observation that severe neurological PRLTS3 phenotypes are caused by mutations that drastically affect the native CLPP protein structure and show that large deletions might be responsible for a substantial part of these CLPP defects.

Whereas symptoms such as SNHL might manifest during early childhood with gradual progression and variable impact, indications for infertility such as impaired sex hormone profiles can only be detected after puberty. Age-dependent symptom presentation and phenotypic heterogeneity can therefore impede genetic diagnosis, especially during early disease onset when only part of the disease manifestations is present. We demonstrate that similarity in brain-MRI pattern of abnormalities among patients can be successfully used to identify other PRLTS3 patients, already at early disease onset. Our selection criteria were signal abnormalities predominantly involving both the subcortical and deep cerebral white matter and the middle blade of the corpus callosum. Multifocal and confluent abnormalities predominantly involving the subcortical and deep cerebral white matter, sparing a periventricular rim, are also seen in l-2-hydroxyglutaric aciduria, Kearns Sayre syndrome, and ‘Leukoencephalopathy with thalamus and brain stem abnormalities and lactate elevation’ and Canavan disease (19, 25). However, involvement of the subcortical and deep cerebral white matter is typically associated with sparing of the corpus callosum or involvement of its outer blade. The combination of abnormalities predominantly involving the subcortical and deep cerebral white matter and the middle blade of the corpus callosum is so specific for PRLTS3 that it allowed us to select patients by MRI criteria only. However, since not all PRLTS3 patients suffer from neurological symptoms, this approach may only apply to severely affected cases.

## Author Contributions

Design and conceptualization of the study were executed by MK, IC, and HS. WES performance, data processing, and protein modeling were done by RS, DH, JV, RK, and BK. *In vitro* experimentation was done by TT, MG, and CB. Patient MRI and clinical data analyses were performed by EH, IC, SR, TS-M, and MK. Contribution to the intellectual content were done by SR, TS-M, MK, IC, and HS. All the authors critically revised and approved the manuscript.

## Conflict of Interest Statement

The authors declare that the research was conducted in the absence of any commercial or financial relationships that could be construed as a potential conflict of interest.
