# Supplementary material for: Specific MRI Abnormalities Reveal Severe Perrault Syndrome due to CLPP Defects
Source: Front Neurol. 2016 Nov 16;7:203. doi: 10.3389/fneur.2016.00203 (PMC5110515; doi:10.3389/fneur.2016.00203)
Supplement: Supplementary file 1 [file data_sheet_1.pdf]

SUPPLEMENTARY

Table S1: Primers used for Sanger sequencing and real-time qPCR

A. Primers for *CLPP* Sanger sequencing

Covered exon	Forward primer (M13 tailed)	Reverse primer (M13 tailed)
**CLPP exon 1 + 2**	5'-*TGTAAAACGACGGCCAGT*GAAGCTGTAGTTCCGCCATC-3'	5'-*CAGGAAACAGCTATGACC*GAGTCAGAAGGGGAGGGAAG-3'
**CLPP exon 3**	5'-*TGTAAAACGACGGCCAGT*CCCAGCCTCCCTTAAAACTC-3'	5'-*CAGGAAACAGCTATGACC*TCTGGTCCCCTGAGTCACC-3'
**CLPP exon 4**	5'-*TGTAAAACGACGGCCAGT*CCTCCCCAGGTTTAGGAGAT-3'	5'-*CAGGAAACAGCTATGACC*GCCCAGTCAGTCCTTCTGTC-3'
**CLPP exon 5**	5'-*TGTAAAACGACGGCCAGT*CCCTGTGAGGGGCTGAC-3'	5'-*CAGGAAACAGCTATGACC*GGAGAAGCCCTGGCTTAGAG-3'
**CLPP exon 6**	5'-*TGTAAAACGACGGCCAGT*AACATTCAGGCCAAAACAGG-3'	5'-*CAGGAAACAGCTATGACC*GTCTGGCAGGCCTCTGG-3'

B. Primers for quantifying *CLPP* mRNA expression by real-time qPCR

Gene/exon	Forward primer	Reverse primer
**CLPP exon 2 + 3 (5' side)**	5'-GCGCGCCTATGACATCTACT-3'	5'- AGAGGAGCTGTGCGATAACAA-3'
**CLPP exon 5 + 6 (3' side)**	5'-CCATCCAGGCAGAGGAGA-3'	5'-AGGGTGGACCAGAACCTTG-3'
**TBP exon 5 + 6**	5'- CACGAACCACGGCACTGATT-3'	5'-TTTTCTTGCTGCCAGTCTGGAC-3'

C. Primers for mtDNA and *CLPP* copy-number determination by qPCR

Gene/exon	Forward primer	Reverse primer
**ND5 (mtDNA)**	5'-GCCTTCTCCACTTCAAGTCAA-3'	5'-TGGGTACAGATGTGCAGGAAT-3'
**B2M (nDNA)**	5'-TGCTGTCTCCATGTTTGATGTATCT-3'	5'-TCTCTGCTCCCCACCTCTAAGT-3'
**CLPP exon 3**	5'-GCCTAAATTCTTCCCCATCC-3'	5'-AGAGGAGCTGTGCGATAACAA-3'
**CLPP exon 4A**	5'-CCTCCCCAGGTTTAGGAGAT-3'	5'-TTGAGGATGTACTGCATCGTG-3'
**CLPP exon 4B**	5'-ATGGGCTCCCTGCTTCTC-3'	5'-GCCCAGTCAGTCCTTCTGTC-3'
**CLPP exon 6**	5'-AACATTCAGGCCAAAACAGA-3'	5'-AGGGTGGACCAGAACCTTG-3'

Table S2: Clinical findings

**Patient**<br>**Year of birth**	**Patient 1.1**<br>**1995**	**Patient 1.2**<br>**1993**	**Patient 2**<br>**1994**	**Patient 3.1**<br>**1989**	**Patient 3.2**<br>**1994**
**Patient and family characteristics**					
Gender	Male	Male	Male	Male	Male
Siblings (affected/unaffected)	1 (pat.1.2) / 1	1 (pat.1.1) / 1	0/1	1 (pat.3.2) / 1	1 (pat.3.1) / 1
Consanguinity of the parents	Yes, full cousins	Yes, full cousins	Not known	Not known	Not known
**History**					
Pregnancy/delivery	Premature	Nl/breech delivery	Nl	Premature	Nl
Neonatal period	Feeding problems	Feeding problems	Irritable	No information	Nl
Initial psychomotor development	Delayed	Delayed	Delayed	Delayed	Nl
Unsupported walking (years)	14 months	14 months	15 months	Unknown	16 months
Highest motor milestone	Walking without support	Walking without support	Walking without support, riding bike with training wheels	Walking without support	Walking without support
**Clinical picture**					
Age at presentation	1 year	16 mths	1 year	4 years	3 years
Clinical signs	Congenital deafness, psychomotor retardation, autism, behavioral problems, epilepsy, height and weight < 2SD hernia inguinalis	Deafness from 16 months, psychomotor retardation, autism, behavioral problems, stunted growth, height/ weight <p3,	Deafness noted at age 1 year; mild retardation of initial psychomotor development; stunted growth; developed epilepsy and ataxia at age 3; sensorimotor neuropathy noted at age 16	Deafness without speech development; psychomotor retardation; spastic diplegia; microcephaly, stunted growth; developed epilepsy (photosensitive myoclonic absences) at 11 years; diabetes mellitus at 20 years	Deafness without speech development; psychomotor retardation; spastic diplegia; microcephaly, stunted growth; no epilepsy and EEG Nl;<br>at 20 years hypothyreosis
Disease course	Slow decline	Slow decline	Slow decline, additional episodes of steep decline following illness or stress	Slow decline	Slow decline
Episodes of regression / number	Yes / multiple	Yes / multiple	Yes, more than ten	Not observed	One
Provoking factors	Febrile illness	Viral infections	Viral infection, puberty	-	None identified
Improvement after regression	Yes	Yes, but never back to baseline	Yes, but never back to baseline	-	Yes
Present status	Wheelchair bound, loss of cognitive skills, uses sign language, swallowing problems	Wheelchair bound, loss of cognitive skills, uses sign language, swallowing problems	Wheelchair bound, also loss of fine motor and cognitive skills; uses sign language; GMFCS level V	Wheelchair bound<br>GMFCS level V	Wheelchair bound<br>GMFCS level IV

Patient number<br>Year of birth	Patient 1.1<br>1995	Patient 1.2<br>1993	Patient 2<br>1994	Patient 3.1<br>1989	Patient 3.2<br>1994
**Physical examination**					
Age at latest exam (years)	20 years	22 years	20 years	25 years	21 years
Dysmorphic features	Midface hypo-plasia, thick lips	Coarse facies, hirsutism, diastasis recti, broad short hands	No	Broad face, thick lips, acanthosis nigricans	Broad face, thick lips, acanthosis nigricans
Head circumference	< P3	P 10	Nl, 58cm	P10	P10
Height / weight	<P3 (1.59m) / <P3 50.5 kg)	<p3 (1.60m), <P3 58 kg)	<P3 / <P3	<P3 / P25	<P3 / P50
Internal organs	Nl	Nl	Nl	Nl	Nl
Intelligence	Impaired	Impaired (IQ 50-55)	Impaired	Impaired	Impaired
Vision	0.1	0.5 ri , 0.6 left	Nl	Myopia	Nl
Fundoscopy	Nl	Nl	Nl	Nl	Nl
Eye movements	Nl	Nl	Nl	Strabism	Nl
Nystagmus	No	Nl	Nl	No	No
Ptosis	No	Nl	Nl	No	No
Hearing	Deaf ri 97 and le 90db)	Deaf 108db ri and 103 db le	Deaf	Deaf	Deaf
Dysarthria	Severe	Severe	Severe	Severe	Severe
Dysphagia, tube feeding	Yes/ no	Yes, no	Occasional, no tube	No	No
Arms					
Tone	Increased	Increased	Nl	Increased	Increased
Reflexes	Brisk	Brisk	Nl	Brisk	Brisk
Muscle strength	Wheelchair bound	Generalized paresis	Nl	Nl	Nl
Spasticity	Yes	Yes	Nl	Yes, severe	Yes, severe
Ataxia	Yes, intention tremor, tremor head and neck	Yes, intention tremor, tremor head and neck	No	Yes, dysmetria	Yes, dysmetria
Sensory function	Nl	Nl	Nl	Not tested	Not tested
Extrapyramidal signs	Mild dystonic	Mild dystonic	No	Mildly dystonic	Mildly dystonic
Legs					
Tone	Increased	Increased	Increased	Markedly increased	Markedly increased
Reflexes	Brisk - clonus	Brisk	Brisk	Brisk	Brisk
Babinski signs	+/+	+/+	Not sure	+/+	+/+
Muscle weakness	No	No	No	No	No
Spasticity	+	+	Yes	Yes, more severe than arms	Yes, more severe than arms
Ataxia	+	+	Gait ataxia, when still walking	No	No
Sensory function	Nl	Nl	Decreased, consistent with peripheral neuropathy	Not tested	Not tested
Extrapyramidal signs	Mild dystonia	Mild dystonia	Tremor in legs	Mild dystonia	Mild dystonia
Gait	Spastic-ataxic, uses walker and wheelchair	Spatic-ataxic	Gait ataxia, when still walking	Wheelchair dependent, but at transfers evident spasticity	Wheelchair dependent, but at transfers spastic-ataxic

Nl, normal; -, absent, +, present
